# Homoharringtonine Exerts Anti-tumor Effects in Hepatocellular Carcinoma Through Activation of the Hippo Pathway

**DOI:** 10.3389/fphar.2021.592071

**Published:** 2021-02-24

**Authors:** Haina Wang, Rui Wang, Dan Huang, Sihan Li, Beibei Gao, Zhijie Kang, Bo Tang, Jiajun Xie, Fanzhi Yan, Rui Liang, Hua Li, Jinsong Yan

**Affiliations:** ^1^ Department of Hematology, Liaoning Key Laboratory of Hematopoietic Stem Cell Transplantation and Translational Medicine, Liaoning Medical Center for Hematopoietic Stem Cell Transplantation, Dalian Key Laboratory of Hematology, Second Hospital of Dalian Medical University, Dalian, China; ^2^ Diamond Bay Institute of Hematology, Second Hospital of Dalian Medical University, Dalian, China; ^3^ College of Pharmacy, Dalian Medical University, Dalian, China; ^4^ Division of Hepatobiliary and Pancreatic Surgery, Department of General Surgery, Second Hospital of Dalian Medical University, Dalian, China

**Keywords:** homoharringtonine, hepatocellular carcinoma, apoptosis, hippo pathway, cell-cycle arrest, proliferation

## Abstract

Hepatocellular carcinoma (HCC) is the most prevalent subtype of liver cancer with a mortality rate of approximately 3–6/100,000 and is the third leading cause of cancer-related death worldwide. Although several small-molecule drugs have been developed for the treatment of HCC, the choice of an agent for patients who require systemic chemotherapy at an advanced stage is still limited. The Hippo pathway is an evolutionarily conserved tumor suppressive pathway commonly dysregulated in HCC, which makes it a promising target for anti-HCC therapies. Homoharringtonine (HHT) is an FDA-approved anti-leukemia drug with proven strong anti-tumor activity in solid tumors. In this study, we found that HHT could significantly inhibit HCC cell growth by suppressing cell proliferation and colony formation. Moreover, HHT repressed cell invasion and migration remarkably. Additionally, HHT induced cell cycle arrest at S phase and promoted apoptosis. Most importantly, we showed that HHT-induced apoptosis was a consequence of the Hippo pathway activation. Consistently, the MST1/2 inhibitor, XMU-MP-1, could restore cell viability and reverse HHT-induced cell apoptosis. Furthermore, *in vivo* results confirmed the tumor inhibitory effect of HHT. Taken together, our findings suggest that HHT is a potential alternative therapeutic agent for the treatment of HCC.

## Introduction

Primary liver cancer is currently the fourth most common malignant tumor and the third cause of cancer-related death worldwide (Bertuccio et al., 2017). It includes mainly hepatocellular carcinoma (HCC), intrahepatic cholangiocarcinoma (ICC), and HCC-ICC mixed type, among which HCC accounts for about 85–90% of cases, with a mortality rate of about 3–6/100,000 (Bertuccio et al., 2017). Unfortunately, its incidence and mortality rate have kept rising in the latest decade, despite the advances in preventive measures, screening techniques, and diagnostic and therapeutic technologies (Balogh et al., 2016; Hashim et al., 2016). Besides hepatic resection, liver transplantation and local ablation, transarterial chemoembolization (TACE) combined with radiotherapy, sorafenib, or chemotherapeutic agents (anthracyclines, mitomycin, and cisplatin) is commonly employed for patients with HCC as a liver-directed therapy (Zhao et al., 2016; Kim and Den Abbeele, 2019; Zhao et al., 2020). However, HCC is usually resistant to chemotherapeutic agents in TACE or system chemotherapy (Bruix et al., 2016). Therefore, more efficient new agents should be introduced into the treatment of HCC.

The Hippo pathway is an evolutionarily conserved pathway that was first discovered in *Drosophila*, which plays a pivotal role in organ size control and tissue homeostasis, and orchestrates cell proliferation and cell death (Huang et al., 2005; Dong et al., 2007). The Hippo pathway consists of numerous kinases, including the mammalian Sterile 20-like kinase 1/2 (MST1/2), the MOB kinase activators 1A and 1B (MOB1A and MOB1B), the adaptor protein Salvador homolog 1 (SAV1), the large tumor suppressor homolog 1/2 (LATS1/2), and the end effectors Yes-associated protein (YAP) and WW domain containing transcription regulator 1 (WWTR1/TAZ), which act in a kinase cascade. Upon activation of the Hippo pathway, YAP/TAZ is phosphorylated by the kinase cascade and sequestered in the cytoplasm. By contrast, unphosphorylated YAP/TAZ enters the nucleus, resulting in continuous transcription of the genes involved in cell proliferation (Zhao et al., 2011). In recent years, a tight relationship between Hippo pathway dysregulation and human cancers has been reported (Pan, 2010; Harvey et al., 2013). Notably, HCC occurs in mice with dysfunctional Hippo pathway (Zender et al., 2006), indicating a key role of the Hippo pathway in HCC development. Indeed, more and more researches indicated that the Hippo pathway activity influences liver cell fate, regulates liver tissue growth, regeneration and tumorigenesis (Yimlamai et al., 2015). It’s reported that persistent elevation of YAP was sufficient to dedifferentiate adult hepatocytes into cells bearing progenitor characteristics and induce HCC development, while reduction of YAP by siRNA converted HCC cell to undergo hepatocyte differentiation. (Yimlamai et al., 2014; Fitamant et al., 2015; Zhang and Zhou, 2019).

Homoharringtonine (HHT), extracted from *Cephalotaxus harringtonia* tree and first discovered in China, is now approved by the FDA as a chemotherapeutic agent for acute myeloid leukemia (AML), chronic myeloid leukemia (CML), chronic lymphocyte leukemia (CLL), and myelodysplastic syndrome (MDS) (O’Brien et al., 1995; Feldman et al., 1996; Chen et al., 2011; Jin et al., 2013). HHT induces apoptosis and inhibits cell proliferation via suppressing PI3K/AKT and MAPK/ERK1/2 signaling pathways or suppressing STAT3 activity in various cancer cell lines derived from colorectal cancer, lung cancer, and breast cancer (Kang et al., 2019; Yakhni et al., 2019; Shi et al., 2020). Nevertheless, the effect and potential mechanism of HHT in HCC remain unknown. In this study, we investigated the activity of HHT on cell proliferation, colony formation, cell migration and invasion in HCC cell lines. Furthermore, we examined the molecular signaling pathways involved in mediating the effects of HHT. Collectively, our results indicate that HHT could be an alternative drug for HCC chemotherapy.

## Results

### HHT Inhibits HCC Cell Proliferation and Colony Formation

**FIGURE 1:**
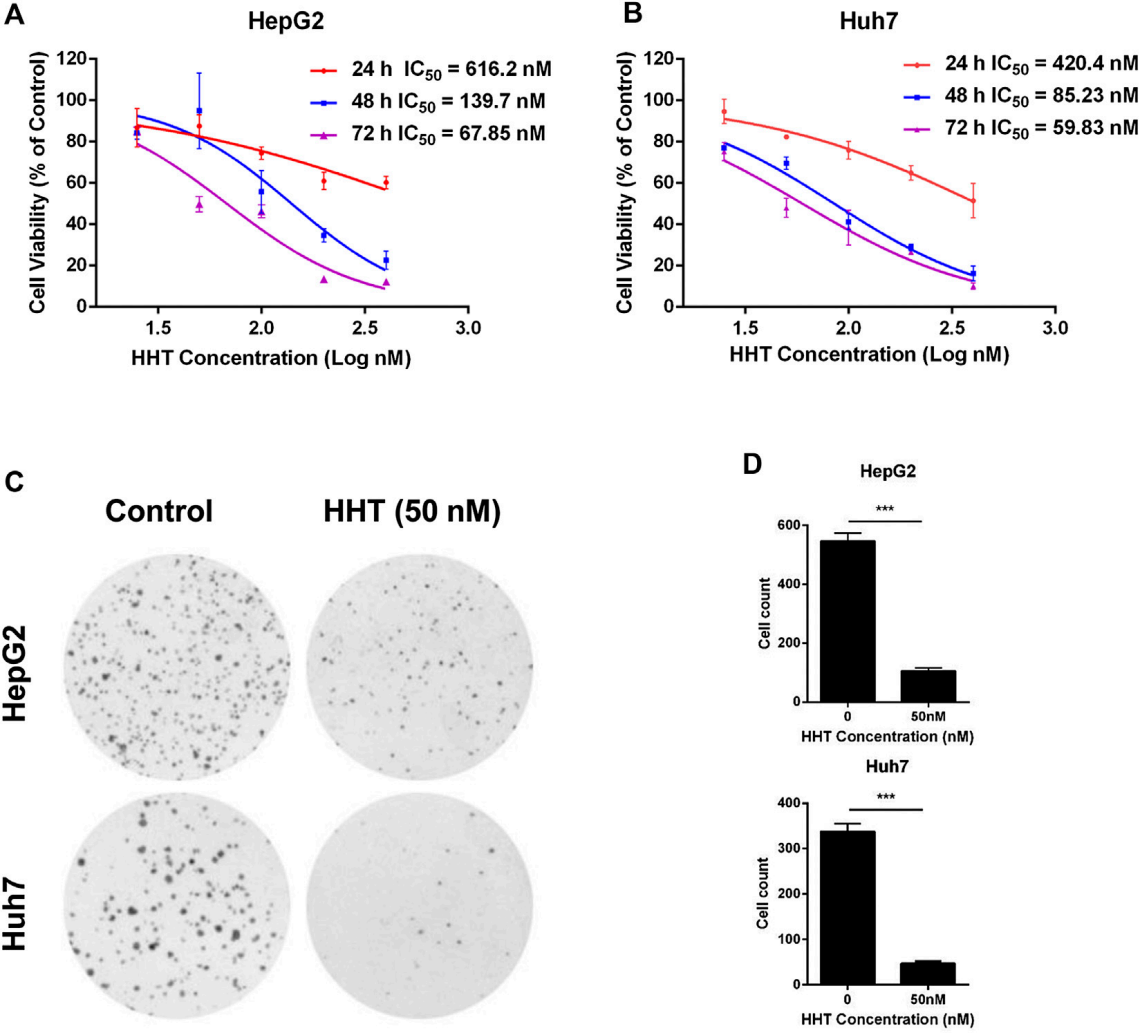
HHT inhibits hepatocellular carcinoma cell proliferation and colony formation. **(A, B)** HepG2 and Huh7 cells were seeded into 96-well plates with 3,000 cells per well, and treated with HHT (0–400 nM) for 24, 48, and 72 h. Cell viability was detected using CCK-8 assay according to the manufacture’s introduction. **(C)** HepG2 and Huh7 cells were seeded into 6-well plates with 1,000 cells per well, and treated with HHT (0 and 50 nM) for 14 days. The colonies were stained with crystal violet and photographed under a Leica DMI4000 microscope. **(D)** Statistical analysis of the colony number in **(C)**. *p < 0.05, **p < 0.01, ***p < 0.001 by one-way ANOVA, followed by Dunnett’s test or Tukey’s test. N = 3. Error bars = S.D.

To clarify the activity of HHT on the proliferation of HCC cells *in vitro*, four HCC cell lines HepG2, Huh7, SMMC-7721 and MHCC-97H were treated with different concentrations of HHT (25, 50, 100, 200, and 400 nM) for 24, 48, and 72 h. CCK-8 assay showed that the cell viability was significantly inhibited by HHT in a dose- and time-dependent manner in the four HCC cell lines (Figures 1A,B; Supplementary Figures S1A,B). Based on the IC_50_ measured, 25, 50, and 100 nM and 48 h were selected for the following experiments as HHT concentrations and timepoint, respectively. To further assess the selectivity of HHT toward HCC cells, we evaluated the toxicity of HHC in a normal hepatic cell line L02 (Supplementary Figure S1C). Interestingly, we found that the HHT toxicity toward L02 is minimal even at a high dose of 400 nM.

Next, the effects of HHT on HCC cell colony formation were tested. Cells were pretreated with or without HHT (50 nM) for 48 h, then cultured for 14 days prior to crystal violet staining (Figure 1C). As shown in Figure 1D, HHT treatment lead to a significant reduction of colony numbers in both cell lines. Taken together, the results demonstrate that HHT suppresses proliferation of HCC cells.

### HHT Attenuates Cell Migration and Cell Invasion

**FIGURE 2:**
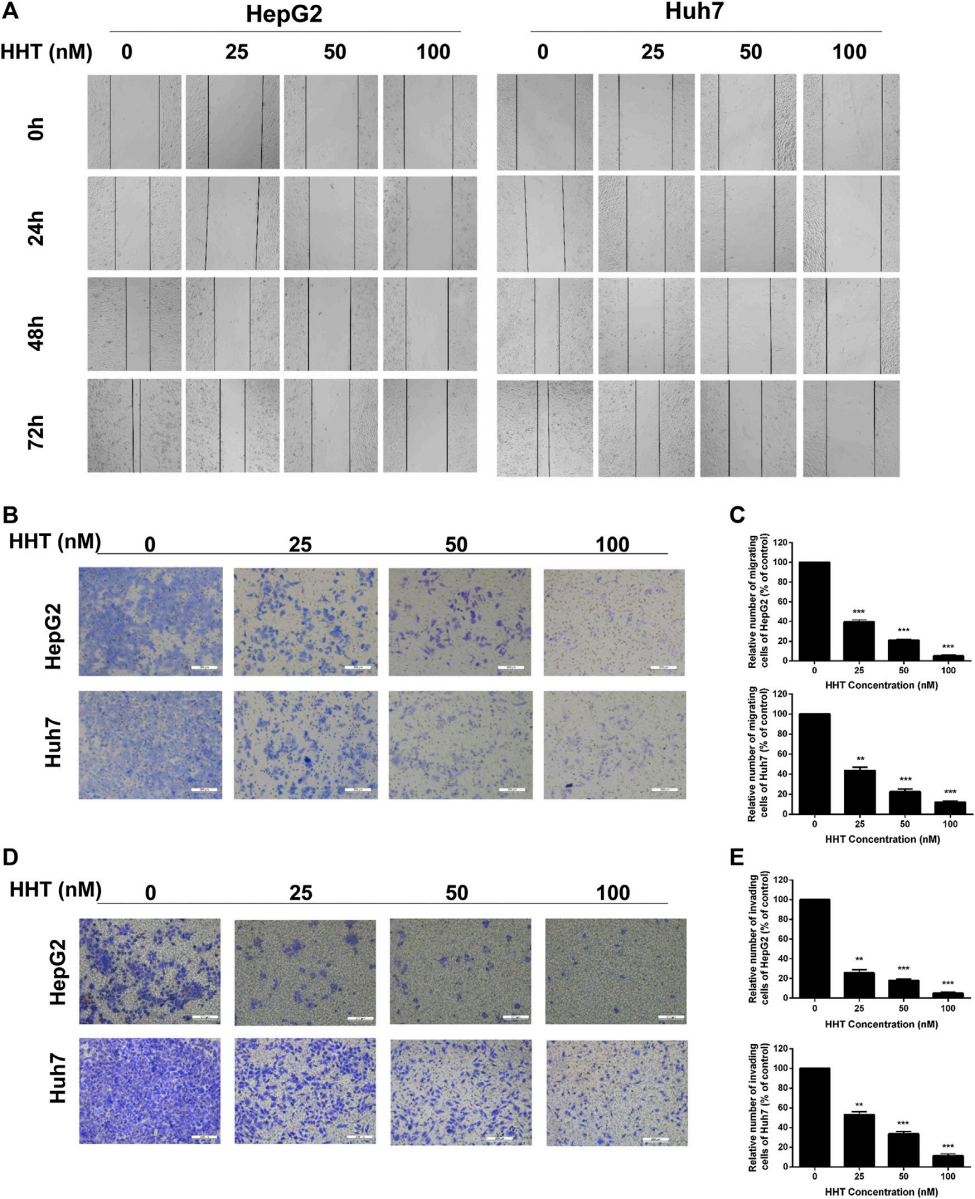
HHT inhibits migration and invasion of hepatocellular carcinoma cells. **(A)** HepG2 and Huh7 cells were seeded into 6-well plates and an injury line was made using a plastic pipette tip. Then the cells were treated with HHT at different concentrations (0, 25, 50, 100 nM) for 24, 48, and 72 h. The gap of the injury lines were photographed and the representative images were displayed. **(B)** Transwell assay was used to evaluate the effects of HHT on cell migration. Representative images of crystal violet stained cells migrating through the transwell insert filter after 24 h are shown. **(C)** Statistical analysis of migrating cell numbers in **(B)**. **(D)** Representative images of crystal violet stained cells invading through the Matrigel after 24 h are shown. **(E)** Statistical analysis of invading cell numbers in **(D)**. Scale bar: 200 μM *p < 0.05, **p < 0.01, ***p < 0.001 by one-way ANOVA, followed by Dunnett’s test or Tukey’s test. N = 3. Error bars = S.D.

Subsequently, wound healing and transwell assays were performed to assess the effect of HHT on HCC cell migration and cell invasion. The wound healing ability of both cell lines gradually decreased in a dose- and time-dependent manner (Figure 2A). Moreover, migratory and invasive capability were both significantly lower in HHT-treated than in control cells (Figures 2B,D), consistent with the results of the wound healing assay. The percentage of invading cells decreased by approximately 60, 80, and 90% in HepG2 cells and 80, 85, and 90% in Huh7 cells, after treatment with 20, 50, and 100 nM HHT, respectively, while the migration rate decreased by about 50, 80, and 90% in both cell lines (Figures 2C,E).

### HHT Induces HCC Cell Cycle Arrest and Apoptosis

**FIGURE 3:**
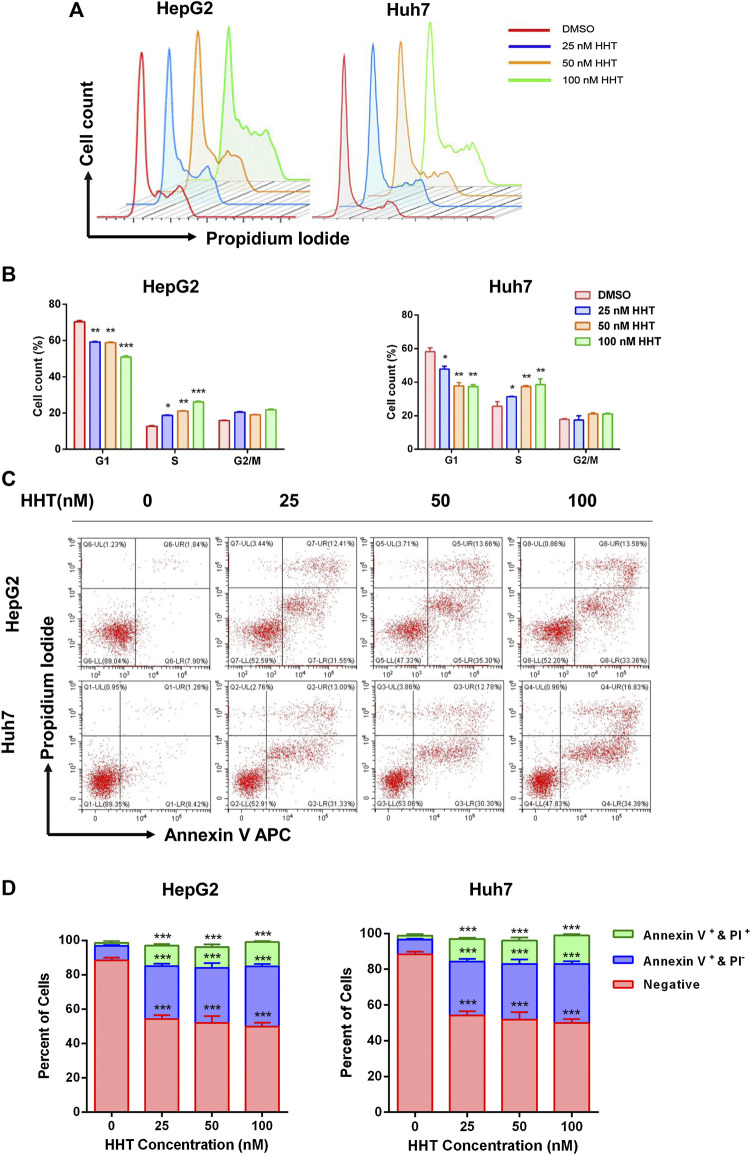
HHT induces S-phase arrest and apoptosis in hepatocellular carcinoma cells. **(A)** HepG2 and Huh7 cells were treated with HHT at different concentrations (0, 25, 50, 100 nM) for 48 h. Cells were harvested, stained with propidium iodide, and analyzed by flow cytometry. **(B)** Statistical analysis of cell numbers in different cell cycles in **(A)**. **(C)** HepG2 and Huh7 cells treated with different concentrations of HHT (0, 25, 50, 100 nM) for 48 h were stained with FITC Annexin-V and propidium iodide. Cell apoptosis were analyzed by flow cytometry. **(D)** Statistical analysis of apoptotic cell numbers in **(C)**. *p < 0.05, **p < 0.01, ***p < 0.001 by one-way ANOVA, followed by Dunnett’s test or Tukey’s test. N = 3. Error bars = S.D.

To further explain the inhibitory mechanism of HHT in HCC cells, cell cycle progression and apoptosis were evaluated using flow cytometry. HepG2 and Huh7 cells were treated with the indicated concentrations of HHT for 48h. HHT treatment led to a gradual decrease in the number of G1-phase cells in parallel to a gradual increase in S-phase cells, in a concentration-dependent manner (Figures 3A,B).

**FIGURE 4:**
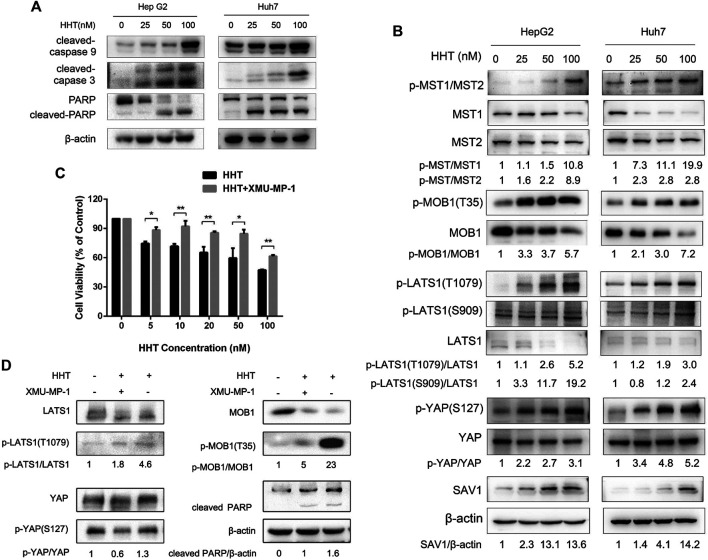
HHT induces apoptosis in hepatocellular carcinoma cells through activating the Hippo pathway. **(A)** Representative western blot results showing protein levels of cleaved caspase 9, cleaved caspase 3, and cleaved PARP in HepG2 and Huh7 cells treated with HHT at the indicated concentration. **(B)** Representative western blot results showing changes in phosphorylation level of proteins in the Hippo pathway, including MST1/2, MOB1, LATS1, SAV1, and YAP, after HHT treatment. **(C)** Huh7 cells pretreated with or without 1 μM XMU-MP1 were cultured with different concentrations of HHT (0, 5, 10, 20, 50, 100 nM) for 48 h; cell viability was then examined by CCK-8. **(D)** Huh7 cells pretreated with or without 1 μM XMU-MP1 were cultured with different concentrations of HHT (0, 50 nM) for 48 h; expression of apoptotic and Hippo pathway proteins was analyzed through western blot.

HHT also promoted apoptosis in a dose-dependent manner. Flow cytometry analysis demonstrated a remarkable increase in the percentage of both early and late apoptotic cells in HHT-treated groups. After treatment with 100 nM HHT, the percentage of apoptotic cells increased from 9.74 to 46.94 and from 9.70 to 51.22 in HepG2 and Huh7 cells, respectively (Figures 3C,D). To investigate whether the apoptotic pathway was activated by HHT treatment, the levels of PARP, caspase 3, and caspase 9 were examined. As shown in Figure 4A, all three proteins were activated by the drug, as illustrated by the HHT concentration-dependent increase in the levels of their cleaved forms. These results indicate that HHT promotes apoptosis in HCC cells by activating pro-apoptotic effectors.

### HHT Suppresses the Malignant Phenotype of HCC Cells via Activating the Hippo Pathway

Owing to the pivotal role the Hippo pathway plays in liver cancer development, we examined whether HHT could affect this signaling pathway. The expression and phosphorylation status of Hippo pathway proteins were analyzed by immunoblots using lysates from HepG2 and Huh7 cells treated with HHT for 48h. HHT treatment increased the phosphorylation levels of almost all key proteins in the Hippo pathway, including MST1 (T183), MST2 (T180), MOB1 (T35), LAST1 (T1079 and S909), and YAP (S127) (Figure 4B). The levels of total SAV1 also increased after HHT treatment. These results indicate that HHT might suppress HCC cell proliferation by activating the Hippo pathway.

Further, HepG2 and Huh7 cells were pretreated with 1 μM XMU-MP-1, an inhibitor of MST1/2, for 24 h before HHT treatment to investigate whether this inhibitor could reverse the block in cell proliferation caused by HHT. XMU-MP-1 indeed ameliorated HHT-induced inhibition of cell proliferation as well as phosphorylation of LATS1, MOB1, and YAP proteins (Figures 4C,D; Supplementary Figures S2A,B). Furthermore, XMU-MP-1 treatment could rescue HepG2 and Huh7 cells from HHT-induced apoptosis by inhibiting the activation of the apoptotic pathway, as indicated by the lower levels of cleaved PARP in the XMU-MP-1-pretreated group than in the non-pretreated group. These data suggest that HHT may induce apoptosis through activating the Hippo signaling pathway in HCC cells.

### HHT Inhibits HCC Cell Growth in Xenograft Models

**FIGURE 5:**
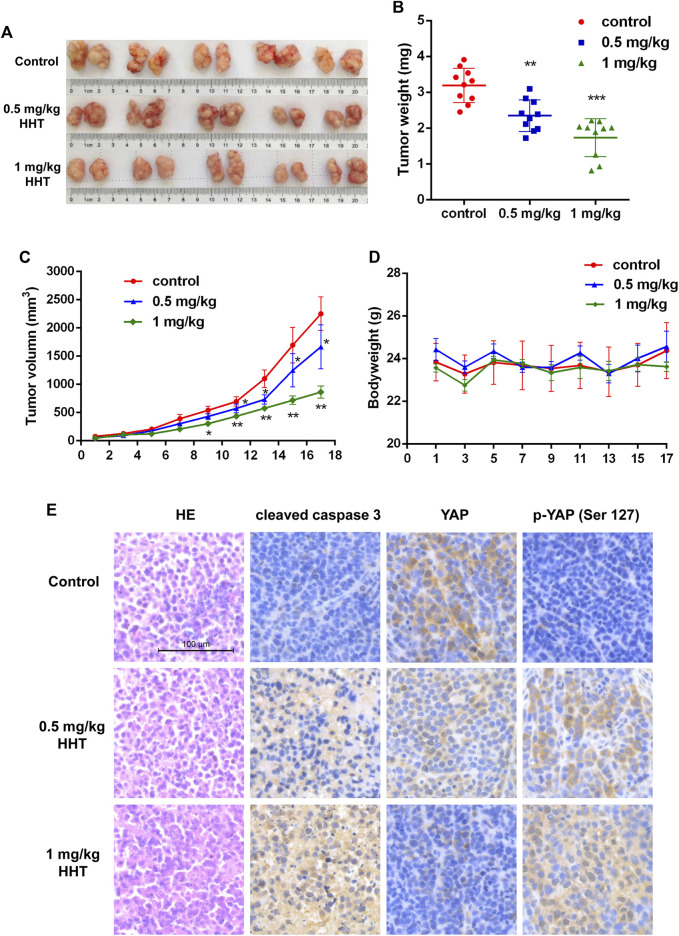
HHT inhibits tumor proliferation *in vivo*. **(A)** Images of tumors from DMSO and HHT-treated mice groups. **(B)** Statistical analysis of tumor weights in **(A)**. **(C)** Statistical analysis of tumor volumes of mice in HHT treatment group and in control group along with time. **(D)** Statistical analysis of the bodyweight of mice in HHT-treated group and in control group along with time. **(E)** Representative images of the HE and immunohistochemical staining of cleaved caspase 3, pYAP (S127), and YAP in tumor sections (×200 magnification), scale bar: 100 μM *p < 0.05, **p < 0.01, ***p < 0.001 by one-way ANOVA, followed by Dunnett’s test or Tukey’s test. N = 3. Error bars = S.D.

To elucidate the potential effects of HHT on HCC growth *in vivo*, we established a subcutaneous HCC model using Huh7 cells in BALB/c nude mice. One week after implantation, mice were separated randomly into three groups with 10 mice per group. DMSO for the control group and different concentrations of HHT for the experimental groups were intraperitoneally injected into the mice every second day for 18days 0.5mg/kg/day and 1mg/kg/day HHT doses were chose according the previous reports (Yakhni et al., 2019; Shi et al., 2020). Remarkably, HHT significantly inhibited tumor growth *in vivo* and decreased the tumor volume (Figures 5A,C). The average tumor weight of mice treated with 0.5mg/kg/day and 1mg/kg/day was approximately 30 and 50% lower, respectively, compared with that of control mice (Figure 5B), whereas the body weight was comparable between HHT-treated and control groups (Figure 5D).

Furthermore, we analyzed the expression of cleaved caspase 3 and the levels of total and phosphorylated YAP. Immunohistochemical staining showed that both cleaved caspase 3 and phosphorylated YAP were increased, while non-phosphorylated YAP levels decreased in HHT-treated mice (Figure 5E). These findings are consistent with the results observed *in vitro*, indicating that HHT could activate the Hippo pathway and apoptotic pathway both *in vitro* and *in vivo*.

## Discussion

HCC incidence has shown a global upward trend over the last decade (Bertuccio et al., 2017), with approximately over 60% patients at an intermediate or advanced stage when newly diagnosed (Yang et al., 2019). For this portion of patients, TACE or systemic treatment combined with targeted molecular inhibitors would yield better outcomes; for instance, multi-targeted tyrosine kinase inhibitors (TKIs) such as sorafenib and lenvatinib have benefited patients with advanced and unresectable HCC (Forner et al., 2018; Yamada et al., 2020; Yoo et al., 2020; Zhao et al., 2020). Due to the shortcomings of chemotherapeutic agents for HCC, for example low response, intolerable side effects, and severe toxicity, it remains necessary to discover a novel agent or an optimized therapy for HCC. It is well known that discovery of a new drug *ab initio* is associated with long development times (more than 10 years), high costs (more than 1 billion US dollars), and, unfortunately, a low success rate (about 4%) (Heinemann et al., 2016), suggesting that repurposing an FDA-approved drug for a new application could be a viable route. Based on the efficacy and safety of HHT for leukemia and its promising anticancer potential in varied solid tumors, such as triple negative breast cancer and colorectal cancer (Yakhni et al., 2019; Shi et al., 2020), we hypothesized that HHT could have antitumor effects on HCC.

To verify our hypothesis, we investigated whether HHT could inhibit HCC cell proliferation and colony formation. We found that HHT could significantly inhibit proliferation of HCC cancer cell lines including HepG2, Huh7, SMMC-7721, and MHCC-97H. The IC_50_ values of HHT in these cells at 48 h were ∼150 nM, 85 nM, 180 nM, and 150 nM, respectively, much smaller than that of sorafenib in these cell lines (∼5 μM) (Liu et al., 2006; Zhang et al., 2019). This suggests that HCC cells are much more sensitive to HHT than to sorafenib, indicating HHT as a promising alternative agent for HCC. Treatment with 50 nM HHT for 48 h also remarkably reduced colony formation in both HepG2 and Huh7 cells, further implying the potential anti-tumor effects of HHT on HCC cells.

Metastasis is one of the hallmarks of cancer, which leads to approximately 90% of cancer-related deaths (Hanahan and Weinberg, 2011). Invasion and metastasis or cancer recurrence due to dissemination are lethal attributes of HCC, resulting in poor prognosis and high mortality (Hartke et al., 2017; Forner et al., 2018). Here, we showed that HHT could strongly inhibit HCC cell migration and invasion in a time and dose-dependent manner.

Several clinically used chemotherapeutics exert their antitumor activity via inducing cell cycle arrest and promoting apoptosis (Ravi et al., 2005; Kim et al., 2009). In our study, HHT treatment lead to cell cycle arrest in S phase in both HepG2 and Huh7 cell lines, which was different from previous reports showing that HHT could arrest the cell cycle in G1/G2 phase in leukemia cells (Mai and Lin, 2005; Wang et al., 2009), indicating an alternative underlying mechanism. HHT also induced apoptosis in a concentration-dependent manner and activated pro-apoptotic proteins caspase 3, caspase 9, and PARP. Together, these data suggest that HHT inhibits HCC cell proliferation by triggering cell cycle arrest and apoptosis.

While normal cells keep the pro-apoptotic and anti-apoptotic signaling balanced, tumor cells can escape apoptosis and sustain unlimited proliferation (Hanahan and Weinberg, 2011). The Hippo pathway is one of the suppressor signaling pathways that cancer cell always evade. Hippo signaling coordinately regulates cell proliferation and apoptosis through post-translational modification of YAP, a mammalian transcriptional coactivator (Huang et al., 2005). Phosphorylation, the main post-translational modification of YAP, is the hallmark of Hippo pathway activation, which promotes ubiquitination-mediated degradation of YAP and thereby suppresses tumor cell proliferation (Hall et al., 2010; Barry et al., 2013; Harvey et al., 2013). It has been reported that Hippo pathway was a tumor suppressor signaling which tightly controls liver size by modulating pro-proliferative and anti-apoptotic gene programs (Yimlamai et al., 2015). However, Hippo pathway is always inactivated in HCC (Patel et al., 2017). Reactivation of Hippo signaling by inhibiting YAP activity could convert HCC cell to differentiate and dramatically reduce tumor number and size (Perra et al., 2014).

Considering loss of Hippo-signaling pathway promotes HCC development (Yimlamai et al., 2015), we next evaluated whether HHT could reactivate Hippo pathway in HCC cells. Strikingly, here, we found that the phosphorylation levels of Hippo signaling proteins MST1/2 (T183 and T180), MOB1 (T35), LATS1 (S909 and T1079), and YAP (S127) were all significantly increased after HHT treatment. Simultaneously, the protein levels of SAV1 also increased. However, the mRNA levels of the key Hippo pathway genes, including MST1, LATS1/2, and YAP, were comparable between the HHT-treated and control groups (Supplementary Figures S3,S4). Although the mRNA levels of LATS1 and LATS2 were significantly upregulated in the 100 nM HHT-treated group, they did not significantly change when using lower HHT concentrations (25 nM and 50 nM) that significantly increased the levels of phosphorylated LATS1/2. These findings suggest that HHT may activate the Hippo pathway mainly through post-translational modification of the key effectors rather than through increasing their transcriptional levels. Moreover, XMU-MP-1, a selective inhibitor of MST1/2, could reverse HHT-induced LATS1 and YAP phosphorylation, restore cell viability, and rescue HHT-induced apoptosis. Collectively, these results implicate that HHT may activate the Hippo pathway via promoting phosphorylation of its core proteins.

Finally, experiments in xenograft models revealed that both the tumor volume and tumor weight were significantly decreased after HHT treatment, with little change in the body weight. Notably, we found a remarkable increase in the levels of cleaved caspase 3 and phosphorylated YAP along with a dramatic decrease in the levels of total YAP in the HHT treatment group, corresponding with the results we detected *in vitro*.

However, there are still some limitations in this study. First, it remains unclear whether HHT activates the Hippo pathway by phosphorylating the MST1/2 directly or by targeting the Hippo pathway upstream effectors, such as GPCR signaling (Harvey et al., 2013). Second, the anti-tumor effects of HHT in HCC *in vitro* and in xenograft models can not accurately mimicking its effects in patients. Therefore, further studies should focus on the mechanism of the Hippo pathway activation induced by HHT, and on the efficacy of HHT as an alternative potential chemotherapeutic drug for HCC in clinical trials.

In summary, here, we evaluated the effects of HHT in HCC and delineated its underlying mechanism *in vitro* and *in vivo*. We demonstrate that HHT could significantly inhibit cell viability, colony formation, and cell invasion and migration of HCC cells. Moreover, HHT could induce HCC cell cycle arrest at S phase and promote apoptosis through activating the Hippo pathway. Our results strongly suggest that HHT is a promising chemotherapeutic drug for HCC treatment.

## Materials and Methods

### Cell Culture

Cell lines HepG2, Huh7, SMMC-7721 were maintained in DMEM high-glucose medium (Hyclone), and MHCC-97H, L02 in RPMI-1640 medium, with 10% fetal bovine serum (FBS, ExCell Bio, China), 100 U/mL penicilin and 100 U/mL streptomycin in a humidified incubator at 37 °C with 5% CO_2_.

### Antibodies and Reagents

Antibodies against caspase 9 (sc-56077) and β-actin (sc-8432) were obtained from Santa Cruz Biotechnology, Texas, United States. Others were purchased from Cell Signaling Technology: anti-PARP (#9542), anti-cleaved caspase 3 (#9661), anti-MST1 (#3682), anti-MST2 (#3952), anti-p-MST1(T183)/MST2(T180) (#49332), anti-LATS1 (#3477), anti-p-LATS1(S909) (#9159), anti-p-LATS1(T1079) (#8654), anti-MOB1 (#13730), anti-p-MOB1 (T35) (#8699), anti-SAV1 (#13301), anti-YAP (#14074), anti-p-YAP (S127) (#13008), and anti-p-YAP (S397) (#13619). RNase A and Propidium Iodide/PI were purchased from Coolaber (Beijing, China) and Sigma-Aldrich (United States), respectively.

### Cell Proliferation Assay

HepG2, Huh7, MHCC-97H, SMMC-7721, and L02 cells were seeded into a 96-well plate with 3,000 cells per well, and treated with HHT at concentrations of 25, 50, 100, 200, and 400 nM for 24, 48, and 72 h. CCK-8 (Beyotime, China) was added to the wells and incubated in the cell incubator for 3 h. The absorbance at 450 nm was measured with a microplate reader (BioTek Elx800).

### Colony Formation Assay

Cells were treated with DMSO or 50 nM HHT for 48 h. Then, cells were harvested, and 1 × 10^3^ cells were plated into each well of a 6-well plate. After 14 days, cells were fixed with 4% paraformaldehyde, and stained with 0.5% crystal violet (Beyotime, China). Colony numbers were counted and colonies were photographed under a Leica DMI4000 microscope.

### Wound Healing Assay

Cells were plated into a 6-well cell culture plate and grown to 70% confluence. An injury line was made using a plastic pipette tip. Different concentrations of HHT were added into the culture medium and the cells were incubated at 37°C with 5% CO_2_ for 24, 48, and 72h. The photographs were acquired at different time points.

### Cell Migration and Invasion Assay

Cells treated with different concentrations of HHT were seeded into the Transwell cell culture inserts (Guangzhou Jet Bio-Filtration, Co., Ltd., China). Transwell inserts were coated with (for invasion) or without (for migration) Matrigel toward the lower compartment and filled with 400 μL of DMEM high-glucose culture medium supplemented with 20% FBS. After incubation for 24 h, Transwell inserts were rinsed with PBS, fixed with 4% paraformaldehyde for 15 min, and stained with 0.5% crystal violet for 15 min. Cells on the upper surface of the filters were removed and cells migrated through the filters were photographed under the microscope at a magnification of ×400.

### Cell Cycle Arrest and Apoptosis Analysis

HepG2 and Huh7 cells were seeded into 6-well plates and allowed to grow to 70% confluence. The culture medium was refreshed and the indicated concentrations of HHT were added. After 48 h incubation, cells were collected and fixed in 70% ethanol at −20 °C for 4 h. The next day, cells were washed with PBS, treated with RNase A (100 μg/ml) for 15min, and stained with PI (50 μg/ml) in the dark for 30min. Cell cycle arrest was detected by flow cytometry. Apoptosis was analyzed using Annexin V-FITC Apoptosis Detection Kit (Beyotime, China) according to the manufacturer’s instructions.

### Western Blot Analysis

Western blot analysis was performed as previously described (Wang et al., 2018). In brief, cells were lyzed in RIPA with cocktail proteinase inhibitor and PMSF; and total cell proteins were separated on a 10% SDS-PAGE and transferred onto a PVDF membrane. The membrane was blocked in the blocking buffer (5% nonfat dry milk in TBST) for 1h at 25 °C, and then incubated with the primary antibodies at 1:1,000 dilution overnight at 4 °C. The next day, the membrane was washed three times with TBST (5 min each time) and then incubated with the HRP-linked secondary antibodies at 1:5,000 dilution for 1h at 25 °C. The proteins were detected by Tanon 4,600 chemiluminescent Imaging system with ECL Western blotting detection kit (Thermo Fisher Scientific).

### Tumor Xenograft Study

Male BALB/c immunocompromized mice (6–8 weeks) weighing from 16 to 20 g were obtained from the Animal Center of Dalian Medical University (Dalian, China). All procedures were performed according to the Institutional Animal Care and Use Committee guidelines and approved by the Institutional Ethics Committee. Huh7 cells (1 × 10^7^) were subcutaneously injected into the right flank of mice. Eighteen mice were randomized into three groups of six mice per group. Vehicle or HHT (0.5 or 1 mg/kg/day) were given via intraperitoneal injection (i.p.) every second day. The mice were sacrificed after 18 days, and tissues were collected for immunohistochemistry analysis.

### Immunohistochemical Analysis

Immunohistochemical analysis was performed according to the standard protocol (Ramos-Vara, 2005). Briefly, the mouse tumor tissues were deparaffinized and rehydrated, the tumor sectionos was submersed in citrate unmasking solution and boiled by microwave for antigen retrieval. Endogenous peroxidase activity was quenched in 3% hydrogen peroxide for 15 min, washed with dH_2_O, and blocked in 5% normal goat serum (Sigma-Aldrich) for 1 h at 25 °C. Primary antibodies against cleaved caspase 3, YAP and pYAP were diluted at 1:400 in 5% normal goat serum and incubated overnight at 4 °C. Anti-rabbit SignalStain Boost detection reagent and SignalStain DAB substrate kit, both purchased from CST, were used according to the protocols. Counterstaining was performed with hematoxylin (CST) and washed with dH_2_O. Sections were dehydrated and mounted with coverslips and mounting medium (CST). Images were captured on randomly selected points on each slide using the Inverted Microscope System at 200 × (Leica).

### Statistical Analysis

Error bars in all figures represent the SD or SEM as indicated in the figure legend. Statistical analyses were performed with SPSS software (version 20.0; SPSS, Inc., Chicago, IL), and graphs were generated using GraphPad Prism 6.0 (La Jolla, CA). All continuous variables were compared using one-way ANOVA, followed by Dunnett’s test or Tukey’s test for multiple comparisons.

## Data Availability

The original contributions presented in the study are included in the article/Supplementary Material, further inquiries can be directed to the corresponding authors.

## Ehics Statement

The animal study was reviewed and approved by the Animal Experimental Ethics Committee of Dalian Medical University.
