# Supplementary material for: Homoharringtonine Exerts Anti-tumor Effects in Hepatocellular Carcinoma Through Activation of the Hippo Pathway
Source: Front Pharmacol. 2021 Feb 24;12:592071. doi: 10.3389/fphar.2021.592071 (PMC7943857; doi:10.3389/fphar.2021.592071)
Supplement: Supplementary file 1 [file datasheet1.docx]

Supplementary Material

# Materials and Methods

## qPCR

Total RNA was prepared from HepG2 and Huh7 cells using the TRIzol reagent (TaKaRa) according to the manufacturer’s instructions. Reverse transcription was performed using the One-Step gDNA Removal and cDNA Synthesis SuperMix (Transgen). qPCR was carried out using SYBR Green Pro Taq HS qPCR kit (Accurate Biotechnology) and the reaction was the following: 30 s at 95 °C, followed by 40 cycles of 5 s at 95 °C and 30 s at 60 °C. The primer sequences used for qRT-PCR are shown in Table 1.

# Supplementary Figures and Tables

## Supplementary Tables

**Table 1.** qPCR primers used in this experiment.

| Gene | Forward Primer (5’-3’) | Reverse Primer (5’-3’) |
| --- | --- | --- |
| β-actin | GAGCGCGGCTACAGCTT | TCCTTAATGTCACGCACGATTT |
| YAP | TGCTGTCCCAGATGAACGTC | AGGGTCAAGCCTTGGGTCTA |
| MST1 | ACAAATCCTCCTCCCACATTCCG | CACTCCTGACAAATGGGTGCTG |
| LATS1 | TGGTCATATTAAATTGACTGAC | CCACATCGACAGCTTGAGGG |
| LATS2 | TAGAGCAGAGGGCGCGGAAG | CCAACACTCCACCAGTCACAGA |

## Supplementary Figures


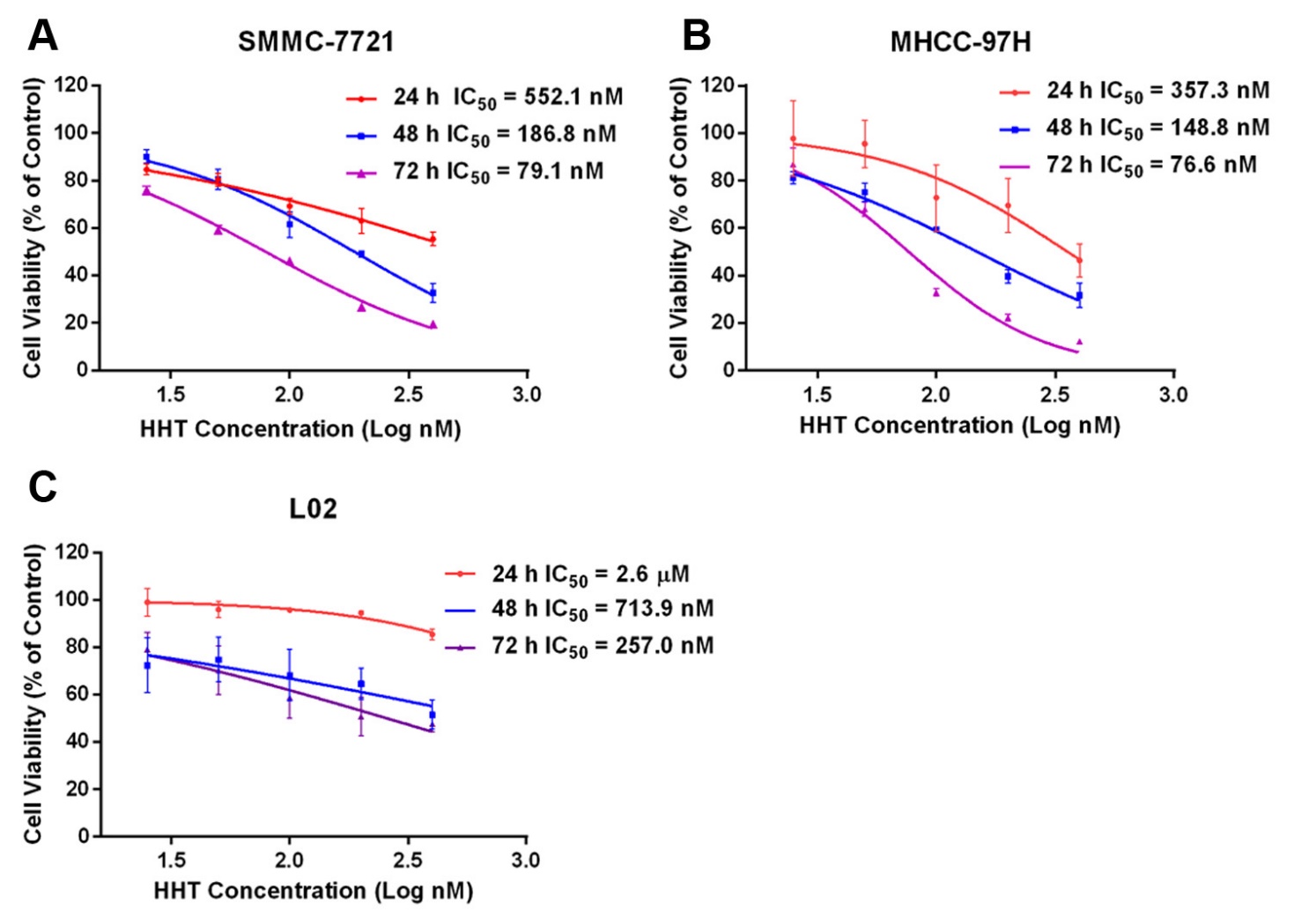


**Supplementary Figure 1.** Effects of HHT on the proliferation of SMCC-7721 (A), MHCC-97H (B) and L02 (**C**) **cells.** SMCC-7721, MHCC-97H and L02 were seeded into 96-well plates with 3000 cells per well, and treated with HHT (25, 50, 100, 200, and 400 nM) for 24, 48, and 72 h. Cell viability was detected using CCK-8 assay. * p < 0.05, **p < 0.01, ***p < 0.001 by one-way ANOVA, followed by Dunnett’s test or Tukey’s test. N = 3. Error bars = S.D.


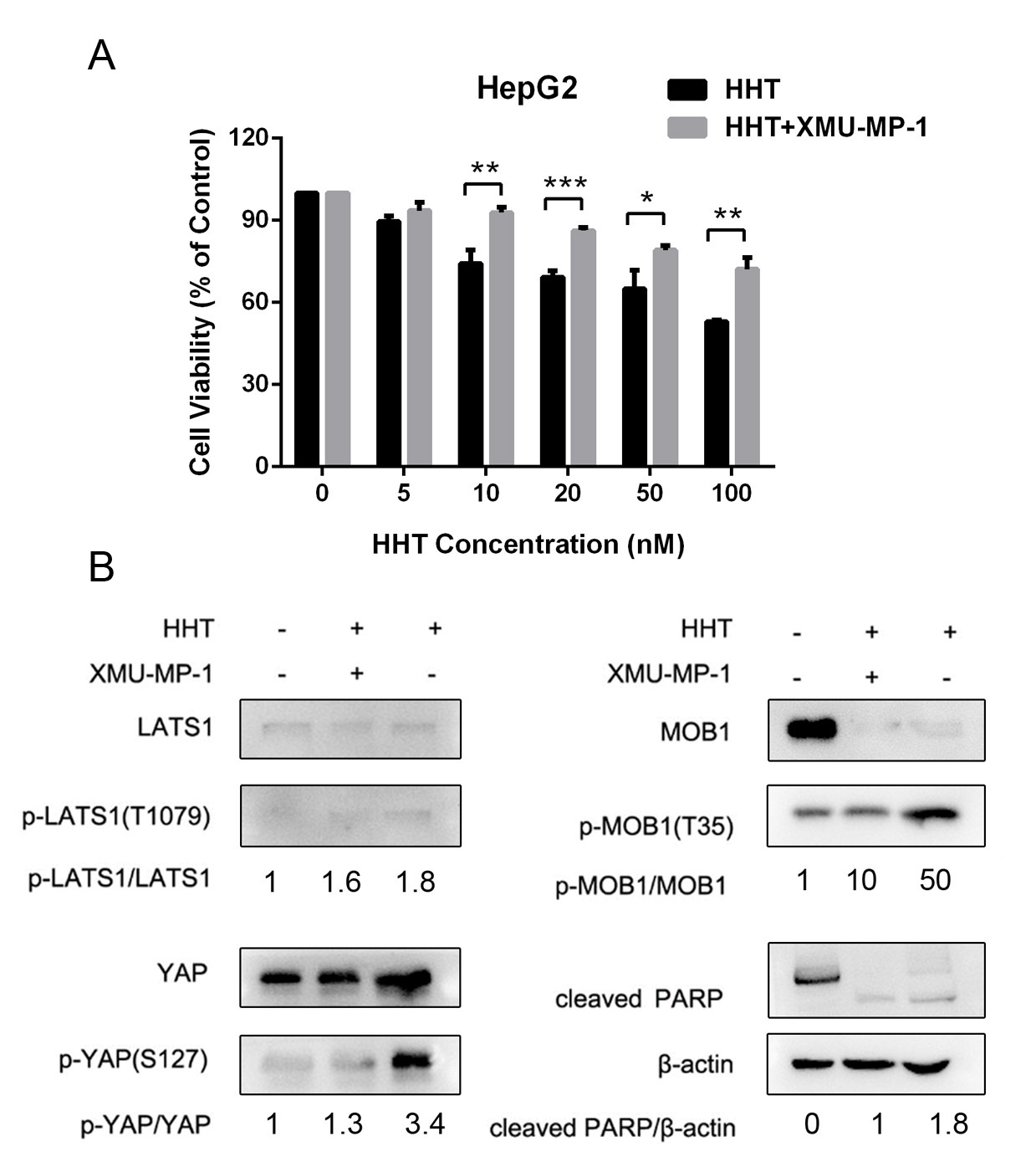


**Supplementary Figure 2. Pretreatment of XMU-MP1 reverses HHT-induced cell apoptosis and Hippo pathway activation. (A)** HepG2 cells pretreated with or without 1 μM XMU-MP1 were cultured with different concentrations of HHT (0, 5, 10, 20, 50, 100 nM) for 48 h; cell viability was then examined by CCK-8. (**B**) HepG2 cells pretreated with or without 1μM XMU-MP1 were cultured with different concentrations of HHT (0, 50 nM) for 48 h; expression of apoptotic and Hippo pathway proteins was analyzed through western blot. * p < 0.05, **p < 0.01, ***p < 0.001 by one-way ANOVA, followed by Dunnett’s test or Tukey’s test. N = 3. Error bars = S.D.


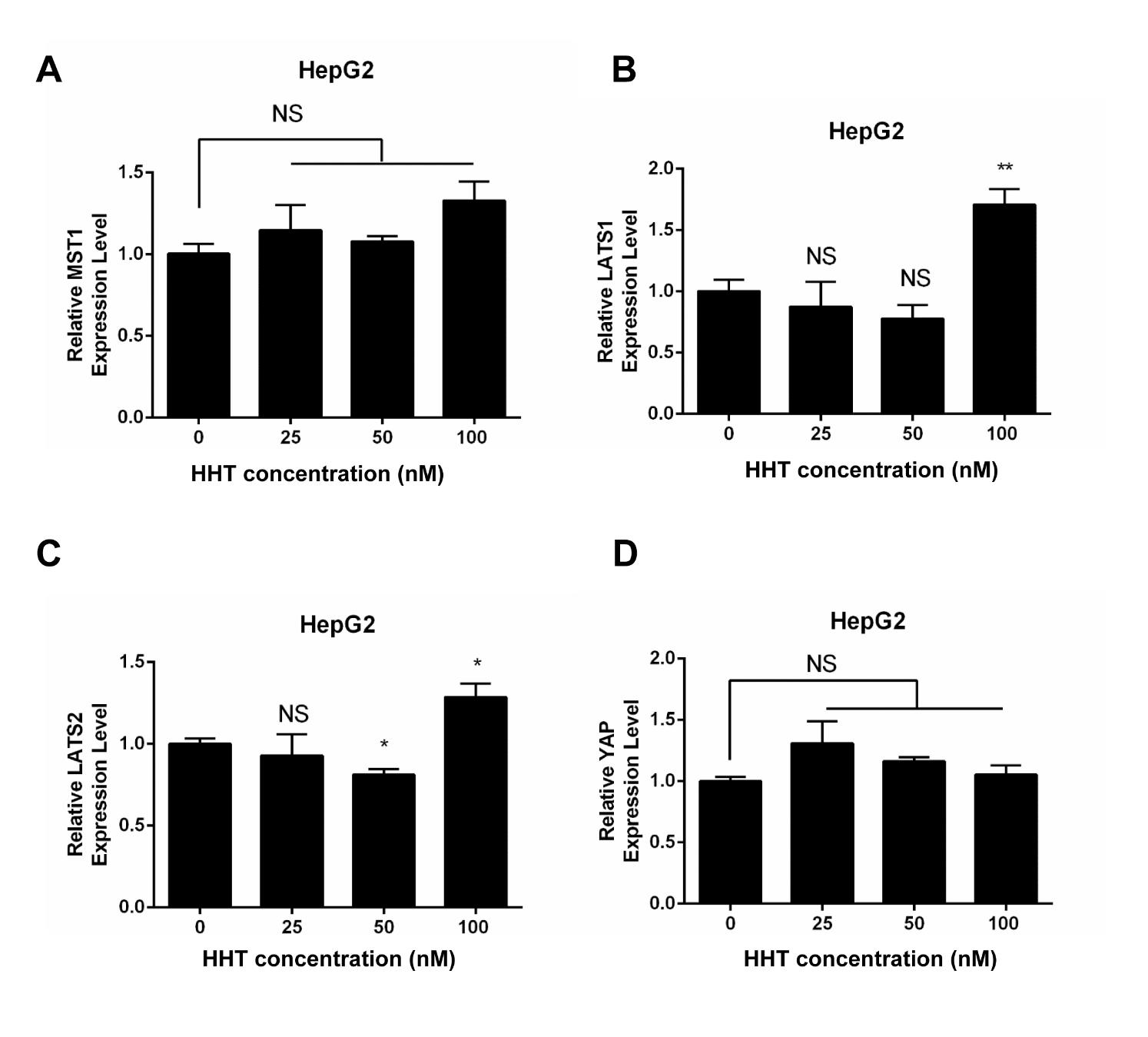


**Supplementary Figure 3.** **Effects of HHT treatment on the mRNA expression level of the Hippo pathway factors in HepG2 cells.** HepG2 cells were treated with different concentrations of HHT (0, 25, 50, and 100 nM) for 48 h. Then, expression of MST1 (A), LATS1 (B), LATS2 (C), and YAP (D) transcripts was examined. * p < 0.05, **p < 0.01, ***p < 0.001 using one-way ANOVA, followed by Dunnett’s test or Tukey’s test. N = 3. Error bars = SEM.


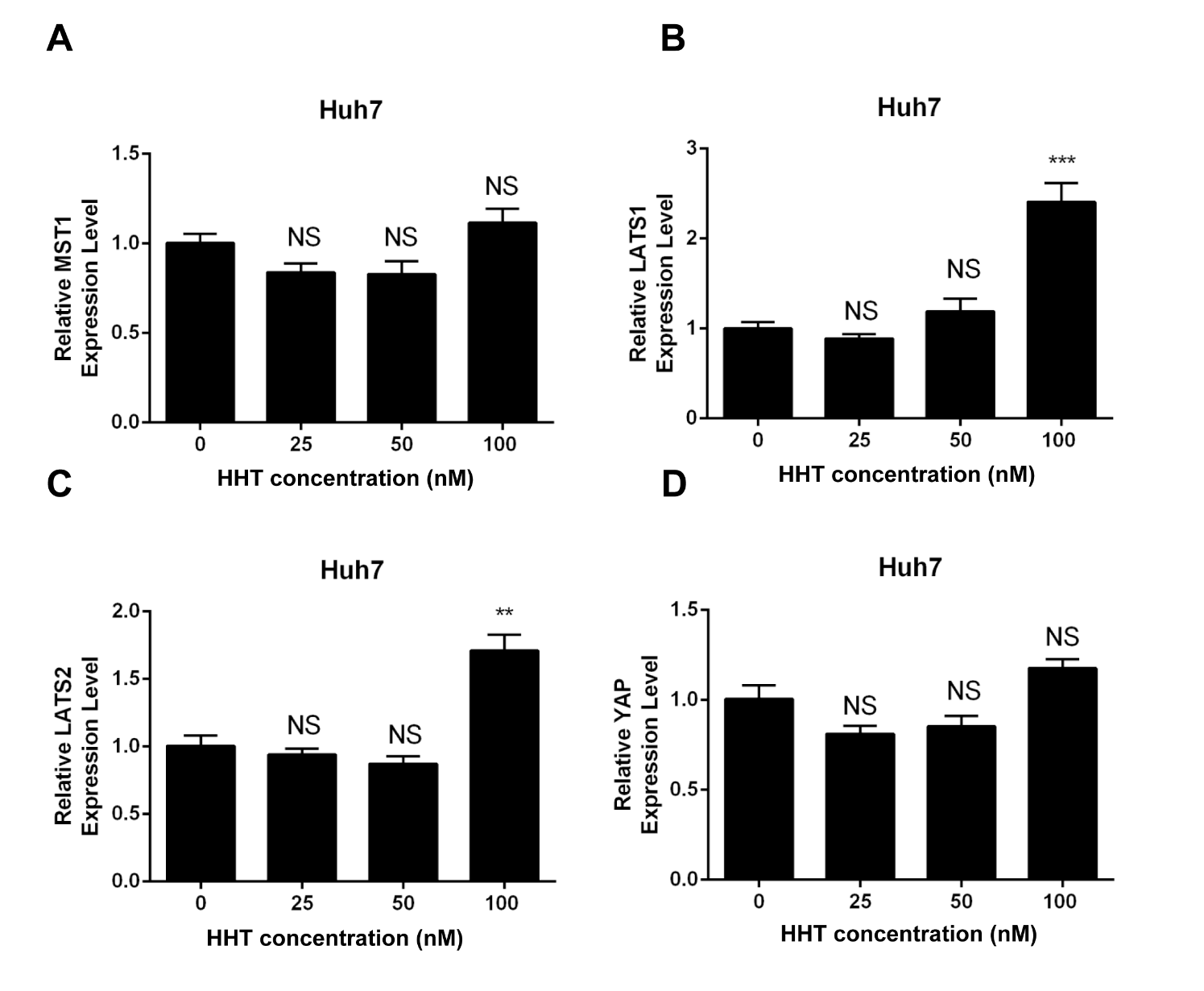


**Supplementary Figure 4. Effects of HHT treatment on the mRNA expression level of the Hippo pathway factors in Huh7 cells.** Huh7 cells were treated with different concentrations of HHT (0, 25, 50, and 100 nM) for 48 h. Then, expression of MST1 (A), LATS1 (B), LATS2 (C), and YAP (D) transcripts was examined. * p < 0.05, **p < 0.01, ***p < 0.001 using one-way ANOVA, followed by Dunnett’s test or Tukey’s test. N = 3. Error bars = SEM.
